# The Ward Floor

**Published:** 1911-01-21

**Authors:** 


### Hospital Architecture and Construction.

[Communications on this subject should be marked "Architecture" in the left-hand top corner of the envelope.]

### THE WARD FLOOR.

### I.—GENERAL CONSIDERATIONS.

Probably few items of construction have caused more thought and discussion in recent years than that of the best material for hospital flooring. Pitch pine and the commoner woods have by general consent been set aside as unsuitable and undesirable. Amongst the essentials in regard to the materials for a successful floor are durability, non-absorbent qualities, an even and uniform surface and the minimum of noise. The latter is dependent largely on the form of construction employed for the base of the floor, but the surface material, if wisely chosen, can be made to contribute to the prevention of avoidable noise.

Another point is the question of whether floors should be covered with linoleum or some other material, or not. It is perfectly certain that, where the best and most modern materials are used for floor surfaces and the construction of the bed of the floor is of the best, any covering of the kind is unnecessary and in our view undesirable. In the case of older hospitals linoleum has been introduced and several hospital authorities hold that it is absolutely unobjectionable. That view is not likely to be ultimately held, however, for when the linoleum or a similar material has been in use for years there must come a time when for hygienic reasons its presence will prove objectionable.

The varieties and types of floors are numerous and in recent years they have multiplied greatly. Now that hygienic efficiency is so essential to success in treatment, the cost and advantages of these various types become matters of the first importance. We shall therefore deal with them a little in detail, and shall be glad to have contributions from medical officers and hospital secretaries, who have paid attention to them and to have their experience of particular floors which may be in use at the hospital or institution under their charge.

A well-known authority says it is acknowledged nowadays that a hospital should, *imprimis*, be of fire-resisting construction; a solid wood floor will fulfil this requirement, but is not practicable owing to the cost. The most economical floor fulfilling the fire-proof requirement is found to be of broken brick or clinker concrete, sand, and cement, with steel joists at about 3-feet intervals; or the still more modern reinforced concrete with iron bars interlacing over and under the joists at somewhat wider intervals; or maybe a fireclay tube floor with iron bands between the tubes. There are many varieties on the market, but the client should be entitled to rely greatly on the architect's advice as to the most suitable type to adopt.

Hospitals have been often designed and built by architects and builders whose education and training has been in domestic work; they naturally bring to the subject of hospital construction the materials and methods of the house, except in cases where medical or surgical specialists direct otherwise. Probably this fact accounts in some measure for the long tolerance of wood-boarded floors; the old-fashioned grooved and tongued and side-nailed flooring gives at once a handsome and hygienic appearance to the ward, and is capable of being polished with beeswax and turpentine to any degree.

*(To be continued.)*

